# Arrhythmias in Neonates and Infants at a Tertiary Care Center

**DOI:** 10.7759/cureus.12861

**Published:** 2021-01-22

**Authors:** Marwan Refaat, Mostafa M Abohelwa, Mohamed Ahmed, Amr Elgehiny, Maryam Ibrahim, Patrick Zakka, Isam El Rassi, Ziad Bulbul, Fadi Bitar, Mariam Arabi

**Affiliations:** 1 Cardiovascular Disease, American University of Beirut, Beirut, LBN; 2 Internal Medicine, Texas Tech University Health Sciences Center, Lubbock, USA; 3 Family Medicine, American University of Beirut, Beirut, LBN; 4 Pediatrics, University of Texas Health Science Center at Houston, Houston, USA; 5 Internal Medicine, American University of Beirut Medical Center, Beirut, LBN; 6 Internal Medicine, Emory University School of Medicine, Atlanta, USA; 7 Cardiac Surgery, American University of Beirut, Beirut, LBN; 8 Pediatric Cardiology, American University of Beirut, Beirut, LBN; 9 Cardiology, American University of Beirut, Beirut, LBN

**Keywords:** neonates, infants, congenital heart disease, arrhythmias

## Abstract

Background

Limited data about arrhythmias in neonates and infants are coming out from the Middle East.

Objectives

To evaluate different types of arrhythmias in neonates and infants at the American University of Beirut Medical Center (AUBMC), a tertiary care center in Lebanon, with the focus on the nature of arrhythmia, treatment modalities and relation to surgery.

Methods

Data were collected retrospectively from the hospital records. We included all neonates and infants presenting to AUBMC between 2013 and 2017. Collected data included: the type of arrhythmia, the treatment modality used and its success, the need for additional modes of treatment, the relationship to congenital heart diseases, and the cardiac surgeries performed.

Results

Of 16,346 subjects admitted to AUBMC between 2013 and 2017, 90 subjects developed arrhythmias that required medical intervention. The most frequent types of arrhythmias were supraventricular tachycardia (62.22%), junctional ectopic tachycardia (13.33%), complete heart block (7.78%), atrial flutter (5.56%), multifocal atrial tachycardia (3.33%), Wolf Parkinson White Syndrome (3.33%), non-sustained ventricular tachycardia (2.22%), sinus pause (1.11%), and premature ventricular contractions (1.11%). Bivariate analysis showed a significant difference between arrhythmias not related to cardiac surgery and arrhythmias related to cardiac surgery in terms of the type of arrhythmia developed, the presence of congenital heart defect, prematurity, and electrolyte disturbances (P-value <0.005). However, multivariate logistic regression showed no significant difference between the two groups after adjustment for the significant variables (P-value > 0.05).

Conclusion

There is a significant difference between arrhythmias not related to cardiac surgery and arrhythmias related to cardiac surgery in neonates and infants at AUBMC. However, the difference disappears after adjusting for different variables.

## Introduction

Arrhythmias in neonates and infants are seen worldwide with an incidence of 24.4 per 100,000 live births, with supraventricular tachycardia being the most common [1]. These arrhythmias might be idiopathic or associated with several risk factors, including congenital cardiac anomalies, surgical procedures, prematurity, immunologic or iatrogenic causes [2]. Some of these arrhythmias may be benign; however, if left untreated, some might lead to thrombi, hemodynamic instability, cardiovascular collapse, or even death, with earlier reports showing infant mortality of 8% [3] to 16% [4] in some cases. Still, benign arrhythmias impose a heavy economic and emotional burden on patient families due to the need for intensive care unit stay, involving 53% of patients as reported in one study [5]. Treatment modalities are various and range from performing simple maneuvers to more complicated pharmacological treatments and pacing [6,7]. Limited data regarding the treatment and management of arrhythmias in neonates (age group 0-29 days) and infants (age group 29 days to 23 months) are coming out from the Middle East with a few studies from Egypt, Turkey, and Iran [8-10]. No available data from Lebanon. Thus, we conducted this retrospective study of arrhythmias in neonates and infants at the American University of Beirut Medical Center (AUBMC).

Our primary objective is to assess different types of arrhythmias arising in this age group, different treatment modalities and their success, second-line treatments, gender differences, and the relationship to electrolyte disturbances and prematurity. Our secondary objective is to study the difference between intrinsic arrhythmias related solely to heart structure and arrhythmias related to interventional procedures performed in this age group.

## Materials and methods

Data source: A hospital-based retrospective cohort study was conducted using the health care records at the American University of Beirut Medical Center (AUBMC), a tertiary care center in Lebanon.

Ethics statement: This study was approved by the Institutional Review Board (IRB) at the American University of Beirut.

Patients: Neonates and infants less than two years of age who developed arrhythmias requiring medical intervention during their hospital admission from January 2013 till December 2017.

Data collection: After subjects were identified, their records were retrieved from the AUBMC database. Relevant data were collected and represented in a table that included the type of arrhythmia, age at diagnosis, gender, treatment modality used, the success of treatment modality used, recurrence of arrhythmia, second-line treatment modality used, recurrence after second treatment modality, congenital heart defect, congenital lupus, familial arrhythmias, prematurity, thyroid status, presentation, relationship to cardiac procedures done whether cardiac catheterization or corrective cardiac surgery, electrolyte disturbances especially potassium, weight and consanguinity between parents. The recorded electrolytes were taken on the morning day of the surgery.

To study the relationship to age, we divided patients into three age categories: less than one month, between one month and one year, and more than one year. The complexity of congenital heart disease was divided into three categories using the American College of Cardiology Task Force 1 of the 32nd Bethesda Conference [11]. This classification included three categories: simple lesions, moderate lesions, and complex lesions. We used the same scheme to categorize our patients. 

Statistical analysis: T-test has been used for continuous variables while chi-square and Fischer tests have been used for categorical variables. P-value < 0.05 was considered statistically significant. Multivariate logistic regression analysis was done to adjust for different variables. Any variable with P-value equal to or less than 0.2 in the bivariate analysis was included in the multivariate analysis.

## Results

Baseline characteristics

Of 16,346 patients below the age of 2 admitted to AUBMC between January 2013 and December 2017, a total of 90 subjects developed an arrhythmia that required medical intervention. Most of these subjects had congenital heart disease (77.78%). The incidence of arrhythmia in this age group was 0.55%. Different forms of arrhythmias were detected, with supraventricular tachycardia (SVT) having the highest incidence (62.22%) followed by junctional ectopic tachycardia (JET) (13.33%), complete heart block (CHB) (7.78%), and atrial flutter (5.56%). Other forms of arrhythmias like multifocal atrial tachycardia, Wolf-Parkinson-White (WPW) syndrome, non-sustained ventricular tachycardia, atrial pause, and premature ventricular tachycardia (PVC) accounted for the rest. None of our patients had abnormal thyroid hormone levels during the time of the arrhythmia. Also, none of them had congenital lupus (Table 1). All patients who developed CHB or JET were CHD patients and had the arrhythmia after the corrective cardiac surgery.

**Table 1:** Descriptive statistics. VT: ventricular tachycardia; SVT: supraventricular tachycardia; PVC: premature ventricular tachycardia; WPW: Wolf-Parkinson-White syndrome; AV: atrioventricular.

	Count	Percentage
Age mean (SD)	7.15 (8.7)	
Age category		
Less than one month	30	33.3
One month to 12 months	37	41.1
One year to two years	23	25.6
Gender		
Female	45	50.00%
Male	45	50.00%
Type of arrhythmia		
Atrial flutter	5	5.56%
AV block	7	7.78%
Sinus pause	1	1.11%
Junctional ectopic tachycardia	12	13.33%
Multifocal atrial tachycardia	3	3.33%
Non-sustained VT	2	2.22%
PVCs	1	1.11%
SVT	56	62.22%
WPW	3	3.33%
Success of first treatment modality used		
No	32	36%
Yes	58	64%
Recurrence after second treatment modality		
No	82	91%
Yes	8	9%
Congenital heart defect		
No	20	22.22%
Yes	70	77.78%
Premature		
No	83	92%
Yes	7	8%
Post cardiac surgery/procedure		
No	30	33.33%
Yes	60	67.76%
Time to develop arrhythmia after surgery (days)		
0 to 5	51	83.6
6 to 10	4	6.55
More than 10	6	9.83
Potassium level		
Hyper	11	12.22%
Hypo	20	22.22%
Normal	59	65.56%
Weight at surgery		
Low	15	16.67%
Normal	75	83.33%
Consanguinity		
No	82	91.11%
Yes	8	8.89%
Complexity		
0	20	22.22%
1	7	7.78%
2	26	28.89%
3	37	41.11%

Association with cardiac surgery

Most of our subjects (60 subjects) developed arrhythmias after cardiac surgery, while 30 of them developed arrhythmias that are not related to any cardiac procedure or intervention, and were related mainly to intrinsic cardiac muscle structure. We studied the difference between the two groups. The type of arrhythmia, prematurity, the potassium level, and the presence of congenital heart disease varied significantly between the two groups, while the difference in age, gender, the success of the first-line treatment, the weight at the time of surgery, and consanguinity showed insignificant difference (Table 2). A multivariate logistic regression analysis model was done to adjust for the significant variables and their association with the outcome, and it showed no difference between the two groups (Table 3). 

**Table 2:** Bivariate analysis between those who had cardiac surgery and those who didn't have any cardiac surgery done (N = 90). *Significant P-value < 0.05 ± Fisher’s Exact test. VT: ventricular tachycardia; SVT: supraventricular tachycardia; PVC: premature ventricular tachycardia; WPW: Wolf-Parkinson-White syndrome; AV: atrioventricular.

	Cardiac surgery		P-value
	Yes (N = 60)	No (N = 30)	
Age (mean ± SD)	6.40 ± 6.99	8.75 ± 9.93	0.252
Age category N (%)			0.489
Less than one month	21 (35.0)	9 (30.0)	
One month to 12 months	26 (43.3)	11 (36.7)	
One year to two years	13 (21.7)	10 (33.3)	
Gender N (%)			1
Male	15 (50.0)	30 (50.0)	
Female	15 (50.0)	30 (50.0)	
Type of arrhythmia N (%)			<0.001*±
Atrial flutter	3 (5.0)	2 (6.7)	
AV block	7 (11.7)	0 (0.0)	
Sinus pause	0 (0.0)	1 (3.3)	
Junctional ectopic tachycardia	12 (20.0)	0 (0.0)	
Multifocal atrial tachycardia	0 (0.0)	3 (10.0)	
Non-sustained VT	2 (3.3)	0 (0.0)	
PVCs	0 (0.0)	1 (3.3)	
SVT	35 (58.3)	21 (70.0)	
WPW	1(1.7)	2 (6.7)	
Premature N (%)			0.005*±
Yes	1 (1.7)	6 (20.0)	
No	59 (98.3)	24 (80.0)	
Success of first treatment			0.276
Yes	41 (68.3)	17 (56.7)	
No	19 (31.7)	13 (43.3)	
Potassium level			0.002*±
Low	18 (30.0)	2 (6.7)	
Normal	39 (65.0)	20(66.7)	
High	3 (5.5)	8 (26.7)	
Weight at surgery			0.765
Normal	51 (85.0)	24 (80.0)	
Low	9 (15.0)	6 (20.0)	
Consanguinity			0.714
Yes	6 (10.0)	28 (93.3)	
No	54 (90.0)	2 (6.7)	
Congenital heart defect			<0.001*±
Yes	60 (100)	10 (33.3)	
No	0 (0)	20 (66.7)	

**Table 3:** Multivariate logistic regression.

	OR	CI for OR		Sig.
		Lower	Upper	
Age by months	.920	.843	1.005	.065
Sex (Ref male)	.429	.089	2.057	.290
Type of arrhythmia	.697	.456	1.065	.095
Premature	.239	.012	4.826	.351

Time from cardiac procedures

**Figure 1:**
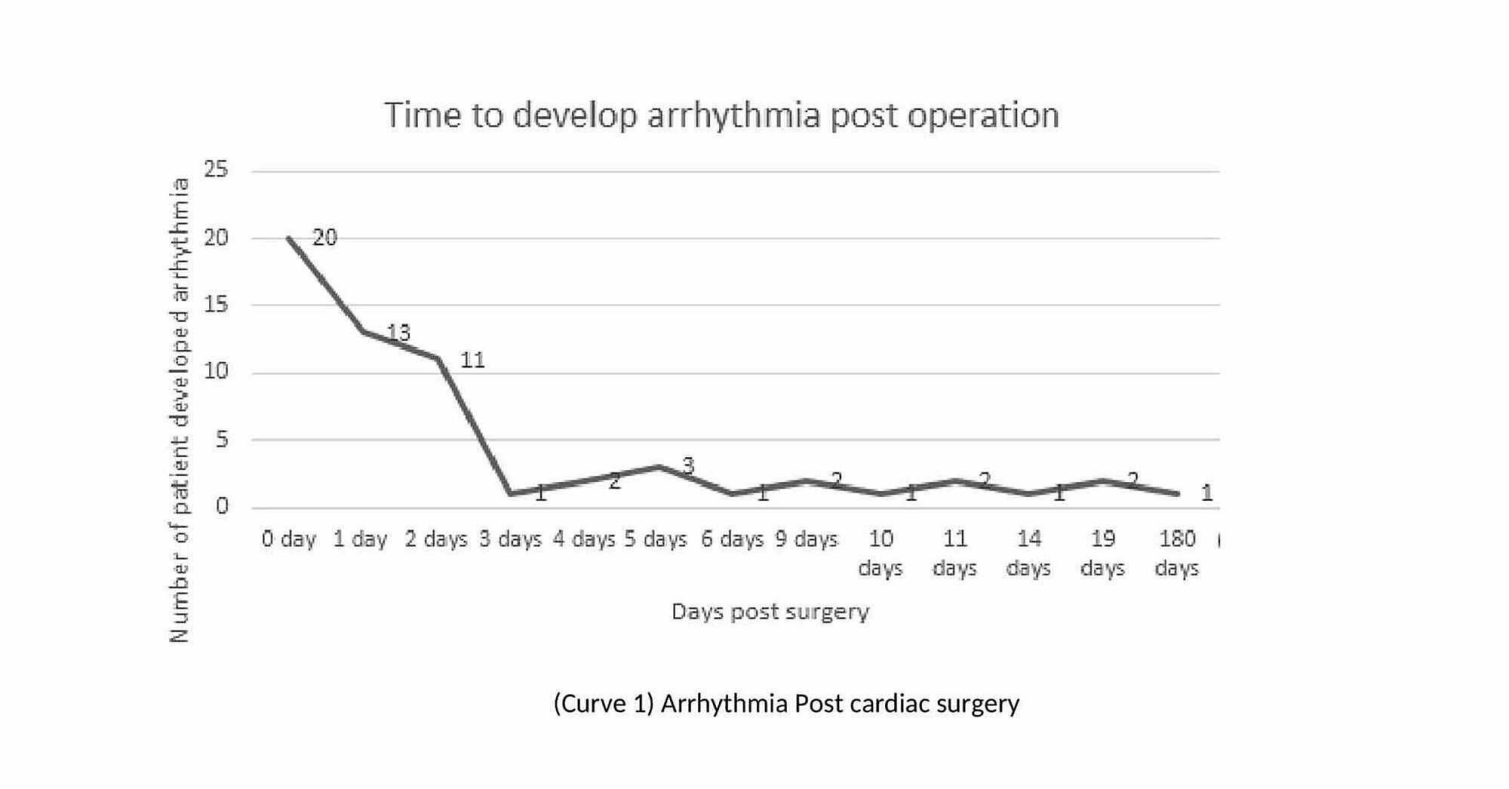
Graph 1: Arrhythmia post cardiac surgery.

Most of our subjects developed arrhythmias in the first few days after corrective cardiac surgeries, with 50 subjects (83.3%) developing the arrhythmia in the first five days (Figure 1).

 

## Discussion

Our data showed the incidence of arrhythmias to be around 0.55 % among neonates and infants less than two years. SVT was the most common (63.3%) among all types of arrhythmia, developing more among CHD patients. SVT developed in 6% of all admitted patients with CHD to our hospital [12]. The increased incidence of arrhythmia among patients with CHD is related to the pathophysiology of their structurally abnormal heart, or to management/interventional procedures such as various medications used to treat the underlying cardiac abnormality or surgical repairs to correct these structural abnormalities [12]. Vagal maneuvers should be attempted first while supplies and personnel are being assembled to proceed to medical therapy, if needed [6,13]. Adenosine is the first-line treatment for the acute management of SVT [6,13]. In our study, we used adenosine as the first-line treatment in all our patients, however, it was successful in 36 patients out of 56 (success was 64%). Amiodarone [14-16] was used as a second-line in 17 patients, followed by DC cardioversion in four of them (only two patients were in shock). Propranolol was used in two patients and flecainide in one patient. The success rate after adding the second-line was 94.6%. Procainamide [14] is described in the literature but not used in our practice. According to these results, we do recommend adenosine as a first-line treatment and amiodarone as a second-line medical treatment for SVT.

The second most common type of arrhythmia in our patients was JET. All patients who developed JET were those with congenital heart disease after undergoing corrective cardiac surgery. The incidence of JET after cardiac surgery in this age population was 1.49 %, which is a little less than its incidence worldwide (5%) [17]. In our practice, we use cooling then amiodarone as the first-line treatment of JET, with a success rate of 75 %. It is reported as the first-line treatment of JET in the medical literature with a success rate of 50-70 % [17,18]. From our experience, we do recommend cooling and amiodarone as the first-line treatment of JET [18].

The third most common type of arrhythmia in this age group was CHB. The incidence of CHB was 0.86% after corrective cardiac surgery, which is nearly the same as the percentage worldwide [19]. All complete heart blocks occurred in patients with CHD after undergoing corrective cardiac surgery. All patients were treated with a permanent pacemaker [20] that was inserted around day 12-14 post cardiac surgery. The success rate was 100%. Despite the presence of rare cases of complete heart block due to other causes such as neonatal lupus [21], we didn't encounter such cases during the period of our study.

The fourth common type of arrhythmia in infants and neonates less than two years was atrial flutter. We found five cases of atrial flutter in this age group during the period of our study. Adenosine was used first for diagnostic purposes. It unmasked the flutter, so DC cardioversion was performed with excellent response and then amiodarone was used for maintenance [22]. DC cardioversion was successful in terminating the arrhythmia completely in all patients. In our practice, we do recommend DC cardioversion as the first-line treatment of atrial flutter in this age category [22]. Despite digoxin being described in the literature [22] as the initial treatment of atrial flutter, it wasn't used in our practice as DC cardioversion has more success rate and was used preferentially for atrial flutter by our cardiology team.

The fifth common type was multifocal atrial tachycardia. We have three cases of multifocal atrial tachycardia with a prevalence of (0.03 %). All three cases were very resistant to therapy, with multiple medications being used including propranolol, amiodarone, flecainide [23], and sotalol [23]. In general, medical treatment of multifocal atrial tachycardia is challenging and uncommon in this age population. Therapy usually is directed at controlling the ventricular response rate with a combination of oral digoxin, beta-blockers, and calcium channel blockers [23]. If not successful, pharmacologic cardioversion is attempted with oral amiodarone, sotalol, flecainide, or propafenone [23]. Therapy usually should be continued for an additional six months to a maximum of one year.

Other rare types of arrhythmias that we encountered in our practice were WPW, PVCs, and non-sustained VT. We have three cases of WPW, two of whom have normal hearts while the other one has congenital heart disease. One case was treated with propranolol that failed to reverse the arrhythmia, so he was given adenosine then flecainide [24]. However, the arrhythmia persisted, so he received DC cardioversion. The patient was then maintained on amiodarone. The other two cases were treated first with adenosine then received DC cardioversion, however, the arrhythmia persisted. As such, one was started on amiodarone and propranolol then discharged only on propranolol. The other had small ASD and was put on amiodarone alone and then discharged on propranolol. Although definitive therapy is catheter ablation [25], none underwent it because our hospital approves it only to patients weighing more than 35 kg.

Moreover, two cases of non-sustained ventricular tachycardia were identified, and both had CHD. Their arrhythmia was effectively terminated with amiodarone [14]. We have one case of symptomatic PVCs [2] in a two-year-old male patient post viral cardiomyopathy that was treated with amiodarone, successfully terminating the arrhythmia. Finally, we encountered one case of sinus pause who was 24 months of age and had a normal heart structure at diagnosis. His past medical history included Thalassemia minor. He presented with syncope and developed syncope five times during his admission, with the Holter monitor showing pauses during the five times. He was treated with a permanent pacemaker.

Since most of our subjects had congenital heart diseases and underwent corrective cardiac surgeries, we opted to study the difference between intrinsic arrhythmias related solely to structural heart disease and arrhythmias related to interventional procedures in this age category. Our subjects were divided into two groups; those who had corrective cardiac surgeries and those who had not. We found that the type of arrhythmia, potassium level, prematurity, and presence of congenital heart diseases varied significantly between the two groups.

Scarce data about neonatal and infantile arrhythmia are coming out from the Middle East. A prospective study by Badrawi et al. [10] from Egypt studied the difference between benign and non-benign arrhythmia in this age population; however, few non-benign arrhythmias were reported (n = 7). SVT, heart block and ventricular tachycardia were the main arrhythmias reported. Another study from Turkey by Sahin et al. [9] included 99 subjects. In this study 36 had common SVT, 27 patients had WPW syndrome, 10 had focal atrial tachycardia (FAT), five had concealed accessory pathway, six had permanent junctional reciprocating tachycardia (PJRT), six had atrial flutter, and one had congenital junctional ectopic tachycardia. The reported results are close to ours' with few differences in the incidence reported. We recommend more studies about the prevalence and the management of arrhythmias in neonates and infants in our region given the burden on healthcare system by these cases.

Limitations

The study was limited by a small number of patients who did not have cardiac surgeries. Also, as the study was done in one center. We do recommend further studies to be done in other centers around the world.

## Conclusions

There is a significant difference between arrhythmias not related to cardiac surgery and arrhythmias related to cardiac surgery in neonates and infants at AUBMC. However, the difference disappears after adjusting for different variables. This should not change the management strategy for the patient if they had surgery.
